# Enhanced brain functional connectivity and activation after 12-week Tai Chi-based action observation training in patients with Parkinson’s disease

**DOI:** 10.3389/fnagi.2023.1252610

**Published:** 2023-10-10

**Authors:** Lin Meng, Deyu Wang, Yu Shi, Zhuo Li, Jinghui Zhang, Hanna Lu, Xiaodong Zhu, Dong Ming

**Affiliations:** ^1^Academy of Medical Engineering and Translational Medicine, Tianjin University, Tianjin, China; ^2^Department of Neurology, Tianjin Medical University General Hospital, Tianjin, China; ^3^Department of Psychiatry, The Chinese University of Hong Kong, Shatin, Hong Kong SAR, China; ^4^Department of Biomedical Engineering, School of Precision Instrument and Opto-Electronics Engineering, Tianjin University, Tianjin, China

**Keywords:** Parkinson’s disease, motor-cognitive intervention, default mode network, restingstate functional MRI, rehabilitation

## Abstract

**Introduction:**

Motor-cognitive interactive interventions, such as action observation training (AOT), have shown great potential in restoring cognitive function and motor behaviors. It is expected that an advanced AOT incorporating specific Tai Chi movements with continuous and spiral characteristics can facilitate the shift from automatic to intentional actions and thus enhance motor control ability for early-stage PD. Nonetheless, the underlying neural mechanisms remain unclear. The study aimed to investigate changes in brain functional connectivity (FC) and clinical improvement after 12 weeks of Tai Chi-based action observation training (TC-AOT) compared to traditional physical therapy (TPT).

**Methods:**

Thirty early-stage PD patients were recruited and randomly assigned to the TC-AOT group (*N* = 15) or TPT group (*N* = 15). All participants underwent resting-state functional magnetic resonance imaging (rs-fMRI) scans before and after 12 weeks of training and clinical assessments. The FCs were evaluated by seed-based correlation analysis based on the default mode network (DMN). The rehabilitation effects of the two training methods were compared while the correlations between significant FC changes and clinical improvement were investigated.

**Results:**

The results showed that the TC-AOT group exhibited significantly increased FCs between the dorsal medial prefrontal cortex and cerebellum crus I, between the posterior inferior parietal lobe and supramarginal gyrus, and between the temporal parietal junction and clusters of middle occipital gyrus and superior temporal. Moreover, these FC changes had a positive relationship with patients’ improved motor and cognitive performance.

**Discussion:**

The finding supported that the TC-AOT promotes early-stage PD rehabilitation outcomes by promoting brain neuroplasticity where the FCs involved in the integration of sensorimotor processing and motor learning were strengthened.

## 1. Introduction

Parkinson’s disease (PD) is a prevalent neurodegenerative disorder characterized by the loss of dopaminergic neurons in the substantia nigra, resulting in a wide spectrum of motor symptoms, such as bradykinesia, rigidity, gait disorders, and instability (Bloem et al., 2021). The alterations in the cortico-basal ganglia networks play a pivotal role in the coordination of cognitive and motor functions, while cognitive impairments significantly influence movement automaticity and motor control ability (DeLong and Wichmann, 2007; Aarsland et al., 2017; Florio, 2018). Motor-cognitive interactive training is crucial for early-stage PD rehabilitation to slow disease progress.

Motor-cognitive interactive rehabilitation has proven to have an impact on neural pathways related to cognitive and motor functions. Attention and executive functions are a set of top-down processes that modulate goal-based movements (Calleo et al., 2012; Bloem et al., 2015). The integration of cognitive training and motor exercise would enhance PD patients’ ability to plan and execute movements while activating brain regions related to memory, attention, and problem-solving (Maidan et al., 2017; King et al., 2020; Johansson et al., 2022). Current studies have reported various motor-cognitive interactive training frameworks that combine motor training with virtual reality, secondary cognitive tasks, or motor imagery (Ferrazzoli et al., 2018; Herold et al., 2018; Ferrazzoli et al., 2020).

Action observation training (AOT) has drawn more and more attention as an effective motor-cognitive interactive training framework that requires participants to observe and imitate specific movements (Caligiore et al., 2017, 2019). Agosta et al. (2017) showed that 4 weeks of AOT with mobility training could reduce the severity of gait freezing, where gait improvement was associated with increased brain activation of the mirror neuron system. The recruitment of cognitive processing within the AOT would lead to the functional reorganization of brain regions in motor control and movement execution (Sarasso et al., 2021). The incorporation of complex inter-limb coordination with AOT may facilitate cognitive-motor interplay (Li et al., 2012; Ferrazzoli et al., 2020). The AOT, incorporating Tai Chi-based continuous and spiral movements, could serve as a novel, effective motor-cognitive training. However, their rehabilitation effectiveness on early-stage PD patients and the underlying mechanisms are unclear.

Resting-state functional MRI (rs-fMRI), as a non-invasive neuroimaging technique, could provide deep insights into the mechanisms of cognitive-motor interplay where functional connectivity (FC) changes can be assessed (Snyder and Raichle, 2012; Soares et al., 2016). The default mode network (DMN) was commonly used in the fMRI analysis and was found to be related to internal mental-state processes (Buckner et al., 2008). The reduced FCs based on the DMN are correlated with executive dysfunction and progressive cognitive decline in PD (Baggio et al., 2015; Thibes et al., 2017). Cognitive training improved functional integration within the DMN in healthy older adults (Cao et al., 2016). Brain changes in the DMN have revealed that aerobic training could modulate brain metabolism in patients with mild cognitive impairment (Porto et al., 2018). Only a few studies have revealed the effect of the AOT on activating the cortical–subcortical region (Agosta et al., 2017; Sarasso et al., 2021).

This study aimed to investigate the rehabilitation effects and the underlying motor-cognitive mechanisms of Tai Chi-based AOT (TC-AOT) training compared to conventional physical therapy. We explored brain FC changes following the 12-week AOT using rs-fMRI. We hypothesized that the TC-AOT, which integrated action observation, motor imagery, and imitation, would be more effective in improving motor and cognitive functions that can be related to network connectivity reorganization of functional brain regions.

## 2. Materials and methods

### 2.1. Participants and clinical measurement

Thirty idiopathic, early-stage PD patients were recruited at the Tianjin General Hospital according to the following inclusion criteria: (1) aged 50–75 years; (2) Hoehn & Yahr (H & Y) stage ranging from 1 to 2.5 while in “on state” (Hoehn and Yahr, 2001); (3) on an anti-parkinsonian medical treatment with a stable daily dose for at least 4 weeks; (4) a Mini-Mental Status Examination score (MMSE) ≥ 24 with more than 12 years of education as the education level needs to be considered to be the strongest noncognitive factor that can affect the performance of MMSE (Folstein et al., 1975; Chen et al., 2016; Opdebeeck et al., 2016); (5) volunteered to participate in the rehabilitative exercise. Exclusion criteria were as follows: (1) other neurological disorders, such as stroke and post-traumatic brain injury; (2) cognitive impairment or dementia; (3) orthopedic problems that affect mobility; and (4) significant head tremor or any contraindications to MRI examination.

Demographic information and clinical assessment scores, including age, gender, disease duration, levodopa equivalent daily dose (LEDD), H & Y stage, and the MMSE score, were collected at enrollment. Thirty participants with PD were finally enrolled and randomly assigned to the Tai Chi-based AOT group (TC-AOT, *n* = 15) or the traditional physical therapy group (TPT, *n* = 15). General motor and cognitive function, balance and posture control capacity, quality of life, and rs-fMRI scan were evaluated at baseline (W0): General motor function was assessed using the Unified Parkinson’s Disease Rating Scale-Part III (UPDRS-III) (Goetz, 2003); Montreal Cognitive Assessment (MoCA) (Nasreddine et al., 2005) assessed global cognitive function; Berg Balance Scale (BBS) (Franchignoni and Velozo, 2005); and the Mini Balance Evaluation Systems Test (Mini-BESTest) (Bloem et al., 2016) were used to objectively determine the participant’s ability to safely balance and maintain posture. All aforementioned clinical measurements were repeated after 12 weeks of training (W12).

All participants provided written informed content, and the study was approved by the Ethics Committee of Tianjin General Hospital. Our trial was registered at www.chictr.org.cn at ChiCTR22000062596.

### 2.2. Intervention procedure

The study was a randomized, assessor-blind exercise trial. Participants were randomized in a 1:1 ratio to the TC-AOT or TPT groups with a block size of 15. To facilitate unbiased group assignment, a concealed allocation procedure was implemented. The allocations were recorded within sealed and opaque envelopes containing computer-generated random numbers, prepared by a research assistant who was intentionally kept blinded to the contents. The researchers who performed the assessments at the time points of W0 and W12 remained blinded to group assignment. Both TC-AOT and TPT groups were conducted in a controlled clinical setting in Tianjin General Hospital under the supervision of a licensed physiotherapist. Participants’ movement execution and safety were closely monitored to ensure consistency and adherence to the prescribed protocols. No participants had engaged in any exercise or physiotherapy before the study. They were instructed not to initiate any new exercise or physiotherapy during the training period.

Both groups received a 1-h training intervention twice a week for 12 weeks. The TC-AOT intervention was administered in a group setting, with each group comprising 2–6 participants. Participants in the TC-AOT group were informed that a training trial consisted of three phases: (1) watched a 60-s video clip containing one specific Tai Chi sequence movement accompanied by voice instruction and concentrated on learning the performance of the actions; (2) imagined themselves performing these actions for 3 min; (3) imitated the Tai Chi actions as precisely as possible. Each training trial took about 5 min. One participant accomplished eight trials in a training session in total, with necessary breaks. Tai Chi movements were selected from the most popular Yang style (Lan et al., 2013). The physiotherapist would systematically progress the intervention based on the participant’s abilities. On the other side, the TPT was administered on a one-on-one basis. The TPT group took a stretching and flexibility exercise consisting of proprioceptive neuromuscular facilitation and gait/balance training with cueing strategies. The therapists followed the same intervention protocol for each participant, but the intervention level was adjusted based on the individual’s conditions. All training programs began with a short warm-up session and ended with a cool-down session.

### 2.3. fMRI analysis

#### 2.3.1. MRI acquisition

Imaging data were captured using a 3.0 Tesla MRI system (GE 3.0 T DISCOVERY MR 750). Foam padding and earplugs were used to minimize head motion and reduce scanner noise. All participants were instructed to hold still and keep their eyes closed without thinking about anything in particular, but not to fall asleep during the period. T1-weighted images were acquired using a magnetization-prepared rapid acquisition gradient echo sequence with the following imaging parameters: repetition time (TR) = 8.2 ms, echo time (TE) = 3.2 ms, flip angle (FA) = 12°, field of view (FOV) = 256 × 256 mm^2^, matrix = 256 × 256, slice thickness = 1.0 mm, no gap, and 188 slices in total. Resting-state fMRI data were acquired using a gradient-echo single-shot echo-planar imaging sequence with the following imaging parameters: TR/TE = 2,000/30 ms, FOV = 220 × 220 mm^2^, matrix = 64 × 64, flip angle = 90°, slice thickness = 3 mm, 36 interleaved transverse slices, and 180 volumes.

#### 2.3.2. Preprocessing of fMRI data

All resting-state functional and structural data were analyzed using SPM12 software (Wellcome Trust Center for Neuroimaging, London, United Kingdom) and CONN toolbox (version 21.a) (Whitfield-Gabrieli and Nieto-Castanon, 2012).^1^ Data preprocessing was conducted using the following procedures: (1) functional images were realigned and unwrapped for head motion correction where data were excluded if the head movements exceeded 1.5 mm/1.5° translation/rotation on any axis (one subjects were excluded due to the exceeded head motion), slice-timing corrected, and ART-based identification of outlier scans for scrubbing; (2) functional and anatomical data were normalized into standard Montreal Neurological Institute (MNI) space using a direct normalization procedure and segmented into grey matter, white matter, and cerebrospinal fluid (CSF) tissues (Ashburner and Friston, 2005; Ashburner, 2007); (3) functional data were smoothed using spatial convolution with a Gaussian kernel of 8 mm full width half maximum (FWHM) and resampled to 2 mm^3^ × 2 mm^3^ × 2 mm^3^; and (4) finally, potential confounding effects including noise components from white matter, CSF, scrubbing regressors, subject-motion realignment parameters, and session effects were included as regressors, and a band-pass filter (0.008–0.09 Hz) was used to reduce low-frequency drift and high-frequency physiological noise in each voxel.

#### 2.3.3. Functional connectivity analysis

FC was processed using a seed-based correlation approach. According to currently available literature (Andrews-Hanna et al., 2010; Hou et al., 2016), the default modes network with 18 priori seeds was selected, including the anterior, dorsal, and ventral medial prefrontal cortices (amPFC, dmPFC, and vmPFC), superior frontal gyrus (SFG), inferior frontal gyrus (IFG), posterior inferior parietal lobule (pIPL), precuneus, posterior cingulate cortex (PCC), anterior temporal lobe (ATL), superior temporal sulcus (STS), temporal parietal junction (TPJ), and hippocampal formation (HF). The MNI coordinates of the 18 seeds are listed in Supplementary Table S1. All seeds were defined as spheres of 6 mm radius with a resolution of 2 mm^3^. Seed-based correlation functional analyses were performed by computing the temporal correlation between each priori seed and the rest of the brain. Fisher’s transformed bivariate correlation coefficients between a seed BOLD time series and each individual voxel BOLD time series were calculated while Fisher’s r-to-z transformed correlation maps were generated. Therefore, an entire brain z-value map was created for each subject.

### 2.4. Statistical analysis

Statistical analysis was performed using SPSS version 26 (SPSS Inc., Chicago, IL). Means and standard deviations were calculated for all demographic and clinical assessment information. Shapiro–Wilk normality test was used to assess the normality of variables. Differences between groups in terms of demographic and clinical data were evaluated by a two-sample *t*-test, Mann–Whitney U test, or chi-square test, as appropriate. Longitudinal changes within groups and the time × group interactions were assessed by using an analysis of variance (ANOVA). The significance threshold was set at *p* < 0.05 (two-tailed).

A group-level analysis of FC was evaluated using a general linear model (GLM). A 2 × 2 mixed analysis of variance (ANOVA) was performed to investigate the main effect of the intervention and the time×group interaction effect. Cluster-level inferences were based on parametric statistics from Gaussian Random Field theory (Worsley et al., 1996) with a threshold setup consisting of a voxel-level (*p* < 0.001) and a family-wise corrected cluster level (p-FWE < 0.05) (Chumbley et al., 2010). For explorational purposes, an uncorrected cluster level *p* < 0.05 was also considered, as the present study aims to investigate any possible effects of intervention (Bender and Lange, 2001).

To further investigate the motor-cognitive interaction mechanism, two-tailed Pearson’s correlation was applied to calculate the correlation between the FC values at the time points W0 and W12, as well as their changes over time within the measured significant clusters and clinical score changes. The significance threshold was set at *p* < 0.05.

## 3. Results

### 3.1. Baseline characteristics and clinical outcomes

Thirty patients were randomly assigned to the TC-AOT group and the TPT group. All patients were in the early stages of PD. Two training groups had no significant difference in all demographic and clinical characteristics, including age, gender, disease duration, LEDD, H & Y stage, and UPDRS-III, as well as in MoCA, MMSE, BBS, Mini-BESTest, and PDQ-39 at baseline. None of the participants received additional physical therapy during the training period. The demographic and clinical data are detailed in Table 1.

**Table 1:** Clinical variables of the two PD training groups at baseline.

Variables	TC-AOT	TPT	*p*
n	15	15	–
Age (years)	65.60 ± 4.53	64.93 ± 6.76	0.75
Gender (F/M)	9/6	7/8	0.54
LEDD (mg)	386.00 ± 162.31	311.05 ± 158.50	0.69
HY (stage)	1.50 ± 0.97	1.60 ± 0.57	0.61
UPDRS-III	15.40 ± 10.21	16.73 ± 13.40	0.34
MoCA	24.27 ± 2.99	24.47 ± 3.48	0.59
MMSE	26.93 ± 2.05	28.07 ± 1.91	0.08
BBS	48.47 ± 2.53	48.20 ± 2.51	0.78
Mini-BESTest	22.87 ± 1.96	23.33 ± 2.50	0.57
PDQ-39	25.00 ± 11.96	21.64 ± 9.15	0.41
After-intervention difference (∆, W12 – W0)			
∆ UPDRS-III	−5.00 ± 5.77^**^	−3.00 ± 3.30^*^	0.35
∆ MoCA	2.10 ± 2.60	−0.20 ± 2.10	0.04^*^
∆MMSE	1.40 ± 2.95	−0.40 ± 1.43	0.10
∆ BBS	2.90 ± 2.18^**^	1.50 ± 1.84^*^	0.14
∆ Mini-BESTest	1.70 ± 1.34^**^	1.30 ± 1.77^*^	0.58
∆ PDQ-39	−18.10 ± 10.78^***^	−9.50 ± 12.28^*^	0.11

Values are mean ± standard deviations. F, female; M, male; LEDD, levodopa equivalent daily dose; UPDRS-III, Unified Parkinson’s Disease Scale; MoCA, Montreal Cognitive Assessment; MMSE, Mini-Mental Status Examination score; BBS, Berg Balance Scale; Mini-BESTest, Mini Balance Evaluation Systems Test; PDQ-39, Parkinson’s Disease Questionnaire-39. *p < 0.05; **p < 0.01; ***p < 0.001.

**Figure 1:**
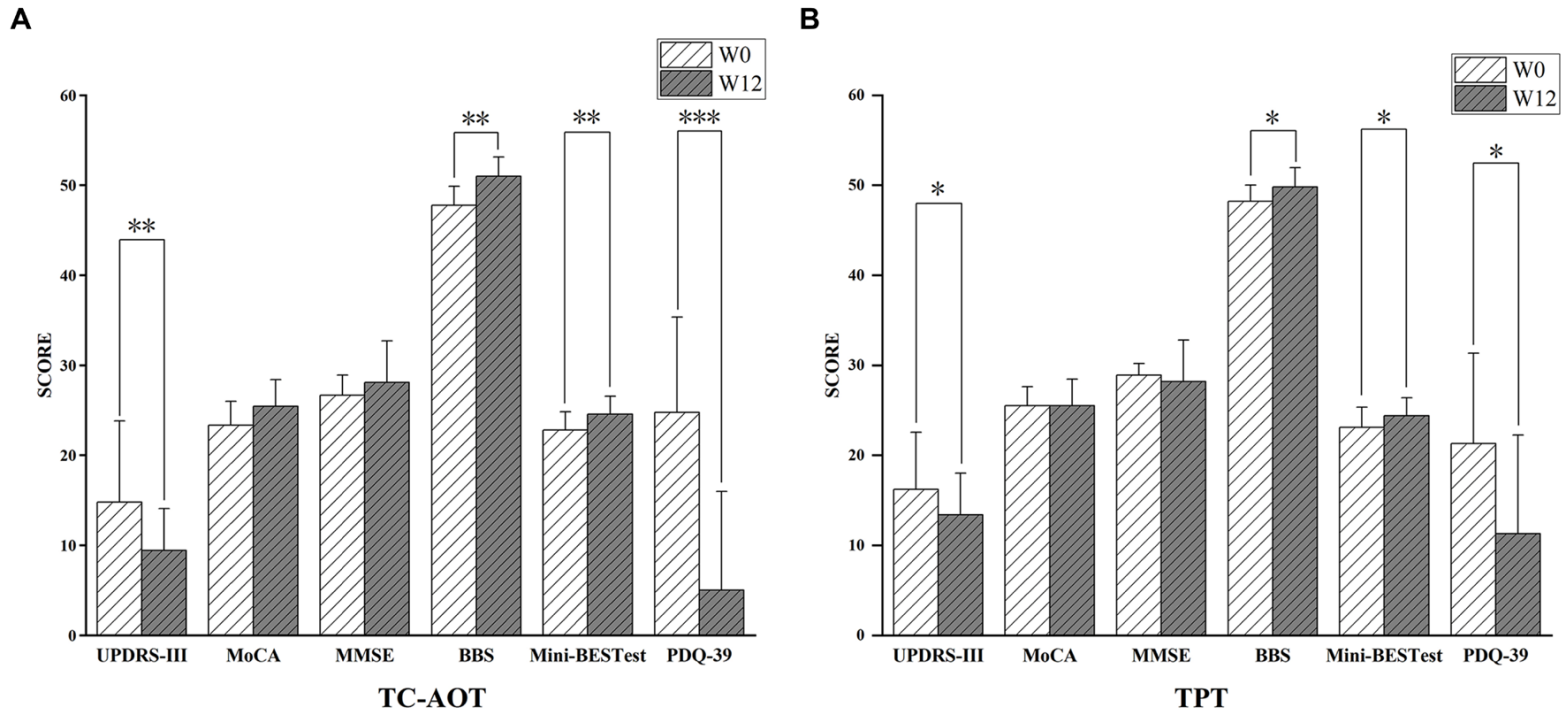
Clinical outcomes after the 12 weeks of TC-AOT and TPT training. **(A)**, Participants in the TC-AOT group showed significantly reduced UPDRS-III scores, improved BBS, Mini-BESTest, and PDQ-39 scores; **(B)**, the TPT group demonstrated decreased scores in UPDRS-III scores, and improvements in BBS, Mini-BESTest, and PDQ-39 scores. UPDRS-III, Unified Parkinson’s Disease Scale; MoCA, Montreal Cognitive Assessment; MMSE, Mini-Mental Status Examination score; BBS, Berg Balance Scale; Mini-BESTest, Mini Balance Evaluation Systems Test; PDQ-39, Parkinson’s Disease Questionnaire-39. *p < 0.05; **p < 0.01; ***p < 0.001.

After 12 weeks of training (W12), both the TC-AOT and TPT groups showed improved mobility with reduced UPDRS-III scores (*p* TC-AOT = 0.008, *p* TPT = 0.022), and enhanced balance performance with greater BBS (*p* TC-AOT = 0.002, *p* TPT = 0.019) and Mini-BESTest scores (*p* TC-AOT = 0.006, *p* TPT = 0.044), as well as a significant improvement in PDQ-39 scores (*p* TC-AOT < 0.001, *p* TPT = 0.030) compared to W0 (Figure 1). There was no significant time × group interaction in clinical assessments between the two groups, except that the TC-AOT group had a greater MoCA score after the intervention while the TPT group exhibited a decreased trend (*p* = 0.04, Table 1).

### 3.2. Seed-based correlation analysis

#### 3.2.1. TC-AOT group vs. TPT group at baseline

There were no significant FC differences were observed at the time point of baseline between the TC-AOT and TPT groups.

#### 3.2.2. Changes within groups after training (W0 vs. W12)

**Figure 2:**
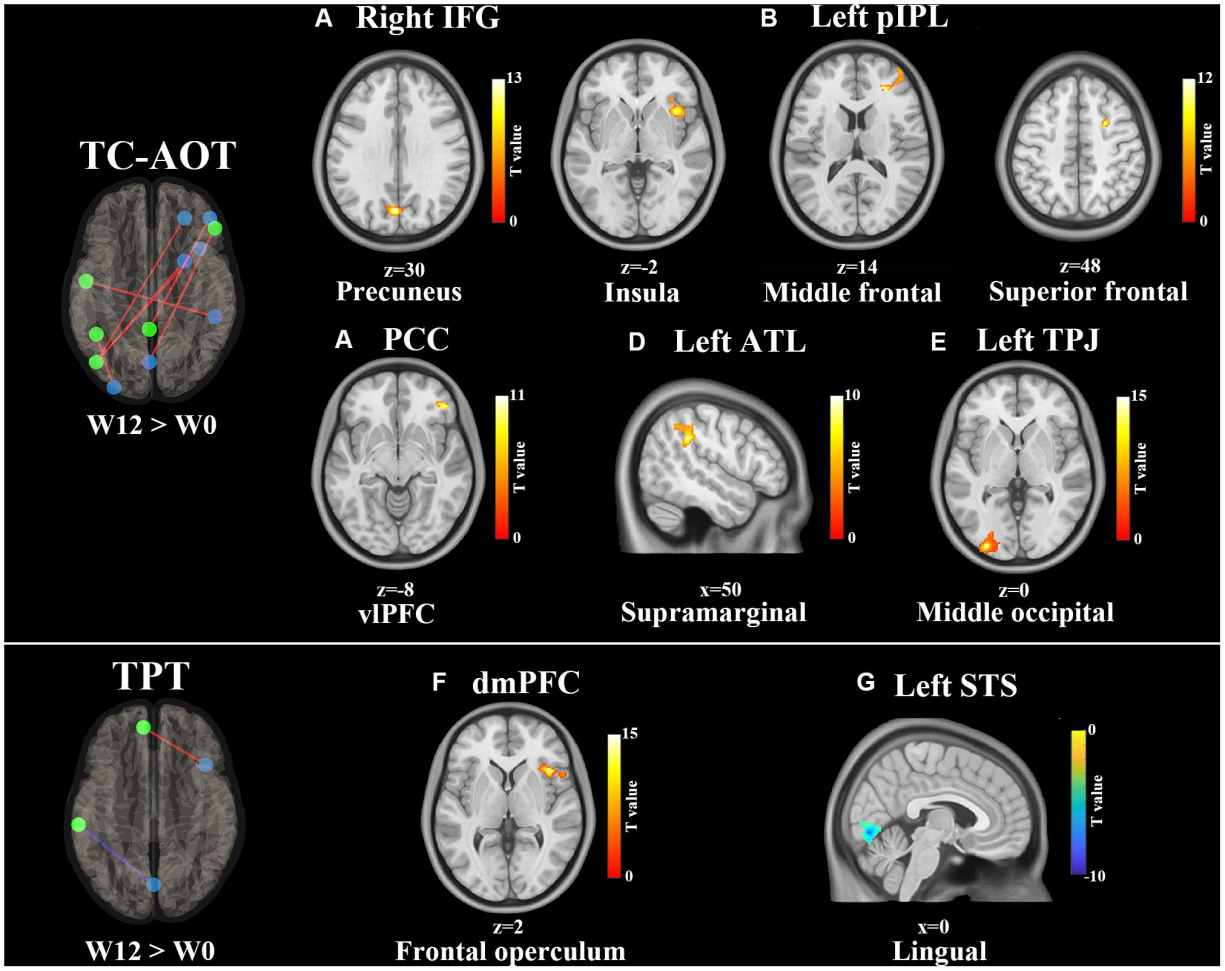
Training effects on FC of the DMN after 12 weeks of intervention (W12 > W0). All FC connections between the DMN seeds and significantly correlated clusters are shown on the left side. Red lines represent the increased FCs, and blue lines refer to the decreased FCs. The results of clusters are shown on axial and sagittal sections of the Montreal Neurological Institute standard brain, where the color bar denotes T values. Increased FC clusters are shown in the hot color and decreased in the cold color. The significance is set at *p* < 0.01 with FWE-corrected. Significant FC changes within the TC-AOT group: **(A)** seed area: right IFG, activated clusters: precuneus; **(B)** seed area: left pIPL, activated clusters: right insula, middle, and superior frontal gyrus; **(C)** seed area: PCC, activated clusters: vlPFC; **(D)** seed area: left ATL, activated clusters: right supramarginal gyrus; **(E)** seed area: left TPJ, activated clusters: middle occipital gyrus. Significant FC changes within the TPT group; **(F)** seed area: dmPFC, activated clusters: right frontal operculum; **(G)** seed area: left STS, activated clusters: right lingual gyrus. Abbreviations: IFG, inferior frontal gyrus; pIPL, posterior inferior parietal lobe; PCC, posterior cingulate cortex; vlPFC, ventrolateral prefrontal cortex; ATL, anterior temporal lobe; TPJ, temporal parietal junction, dmPFC, dorsal medial prefrontal cortex; STS, superior temporal sulcus.

Table 2 shows significant FC changes within the TC-AOT and TPT groups after 12 weeks of intervention. The TC-AOT group exhibited more significant changes in specific brain regions, as shown in Figure 2. The coupling of the right IFG to the left precuneus cortex was strengthened (*p* < 0.001, FWE-corrected) (Figure 2A). Figure 2B shows increased connectivity patterns within the areas including the right insular cortex (*p* < 0.001, FWE-corrected), right middle frontal gyrus (MFG), and Brodmann Area (BA) 10 (*p* < 0.001, FWE-corrected), as well as the right SFG and BA 6 (*p* = 0.002, FWE-corrected), when selecting the left pIPL as a seed (*p* < 0.001, FWE-corrected). Significant active patterns were observed at clusters located at the PCC and right MFG (*p* = 0.003, FWE-corrected) (Figure 2C), as well as the left ATL and the right supramarginal gyrus (*p* < 0.001, FWE-corrected) (Figure 2D). The left TPJ and the left middle occipital gyrus (MOG) also showed an increased connectivity pattern (*p* < 0.001, FWE-corrected) (Figure 2E).

**Table 2:** Significant functional connectivity differences within groups (W12 > W0).

Seed area	Cluster size	Cluster location	MNI coordinate			*p*	*T*
*TC-AOT group, W12 > W0*							
Right IFG							
Cluster 1	177	Precuneus	−2	−74	30	<0.001	11.35
Left pIPL							
Cluster 1	216	Right insula	38	16	−2	<0.001	14.07
Cluster 2	195	Right middle frontal	26	40	14	<0.001	7.84
		BA 10					
Cluster 3	149	Right superior frontal	26	6	48	0.002	14.07
		BA 6					
PCC							
Cluster 1	138	Right vlPFC	46	40	−8	0.003	10.13
Left ATL							
Cluster 1	190	Right supramarginal	50	−38	32	<0.001	9.58
Left TPJ							
Cluster 1	440	Left middle occipital	−30	−94	0	<0.001	16.51

*TPT group, W12 > W0*							
dmPFC							
Cluster 1	214	Right frontal operculum	42	20	2	<0.001	16.63
Left STS							
Cluster 1	185	Right lingual	0	−76	−6	<0.001	−11.01

*p* values are corrected for multiple comparisons by FWE correction. MNI, Montreal Neurological Institute; IFG, inferior frontal gyrus; pIPL, posterior inferior parietal lobule; BA, Brodmann area; PCC, posterior cingulate cortex; ATL, anterior temporal lobe; TPJ, temporal parietal junction; dmPFC, dorsal medial prefrontal cortex; STS, superior temporal sulcus; FWE, family-wise error.

On the other side, the TPT group demonstrated an increased FC between the dmPFC and right frontal operculum cortex (*p* < 0.001, FWE-corrected) and a significantly reduced FC between the left STS and right lingual gyrus (*p* < 0.001, FWE-corrected), as shown in Figures 2F,G.

#### 3.2.3. Changes between groups after training

**Figure 3:**
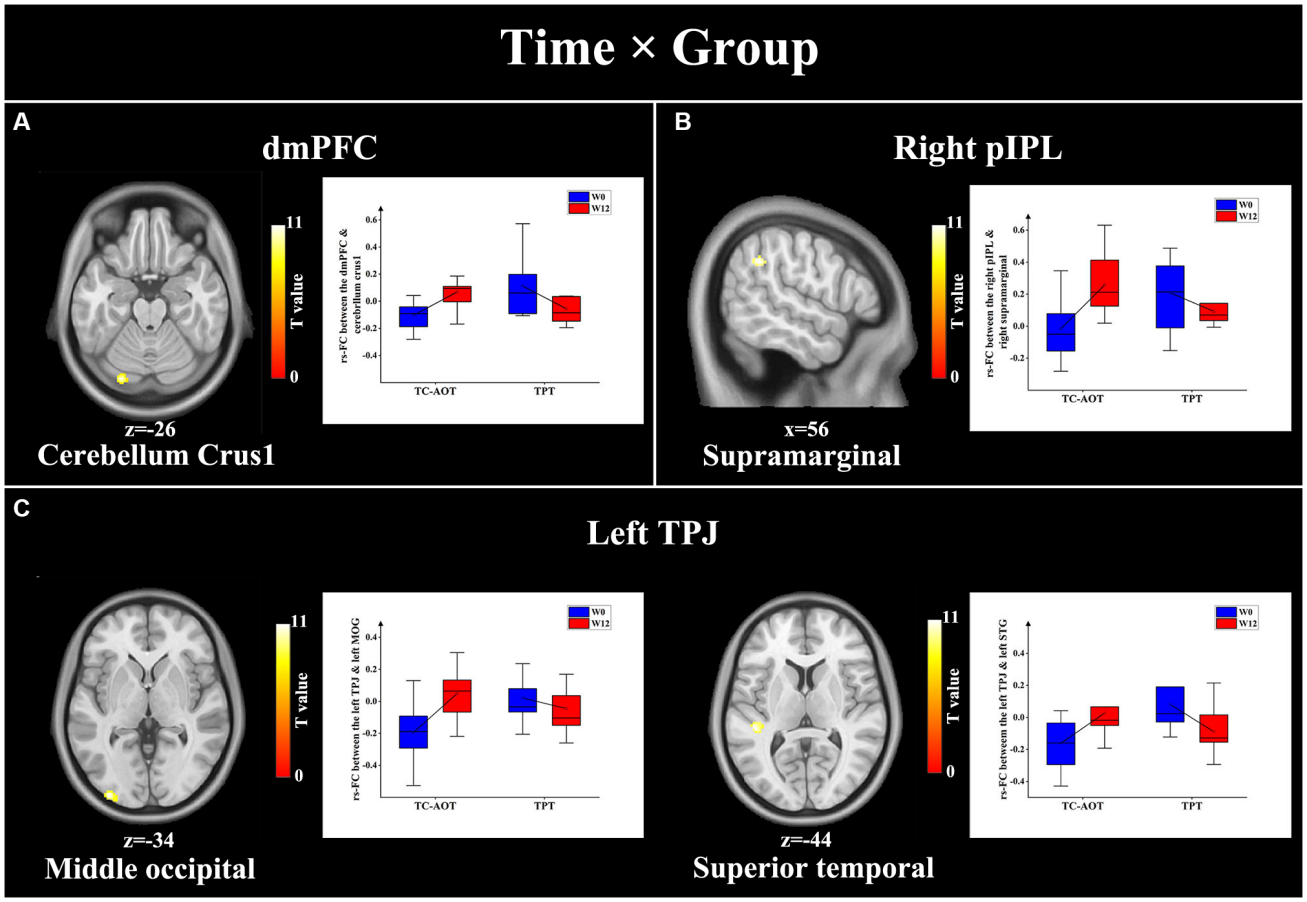
Results of significant time × group interactions between the DMN seeds and specific brain regions. The TC-AOT group exhibited a significant increase in FC values between several DMN seeds and brain regions, whereas the TPT group had decreased FCs. The significance is set at *p* < 0.05 cluster level uncorrected. **(A)** seed area: dmPFC, activated clusters: cerebellum crus1; **(B)** seed area: right pIPL, activated clusters: right supramarginal gyrus; **(C)** seed area: left TPJ, activated clusters: middle occipital gyrus and left superior temporal gyrus. dmPFC, dorsal medial prefrontal cortex; pIPL, posterior inferior parietal lobe; TPJ, temporal parietal junction.

We observed significant time × group interactions between specific DMN seeds and related brain areas, as detailed in Table 3. Compared to the TPT group, the TC-AOT group exhibited increased FC after 12 weeks of training based on different seeds, including the dmPFC to the cerebellum crus 1, the right pIPL, and right supramarginal gyrus as well as between the left TPJ and area of the left middle occipital and superior temporal gyrus (*p* < 0.05, cluster level uncorrected) (Figure 3). There were no other significant time × group interactions when comparing the TPT group to the TC-AOT group.

**Table 3:** Significant functional connectivity differences between groups (W12 > W0) using time × group model.

Seed area	Cluster size	Cluster location	MNI coordinate		*p*	*T*	
*TC-AOT > TPT, W12 > W0*							
dmPFC							
Cluster 1	88	Left cerebellum crus1	−26	−84	−22	0.009	6.21
Right pIPL							
Cluster 1	77	Right supramarginal	56	−46	34	0.017	5.69
Left TPJ							
Cluster 1	117	Left middle occipital	−34	−96	0	0.003	6.79
Cluster 2	59	Right superior temporal	−44	−32	8	0.026	5.75

*p* values are cluster-level uncorrected. MNI, Montreal neurological; dmPFC, dorsal medial prefrontal cortex; pIPL, posterior inferior parietal lobule; TPJ, temporal parietal junction.

### 3.3. Behavioral and neural correlation

Table 4 demonstrates significant correlations between clinical assessment scores and FC changes from seed-based fMRI analysis. In the TC-AOT group, the increased FC between the left TPJ and left MOG after 12 weeks of training was significantly related to the reduction of the UPDRS-III score (*r* = −0.698, *p* = 0.025), while the FC level of the left pIPL and right insula showed a positive correlation with the improvement of the BBS score (*r* = 0.644, *p* = 0.044). The change in FC between the left ATL and right supramarginal was positively correlated with the change in MMSE score (*r* = 0.670, *p* = 0.034). In the TPR group, the FC between the dmPFC and right frontal operculum showed a positive relationship with the enhancement in Mini-BESTest score (*r* = 0.666, *p* = 0.035), and that between the left STS and right lingual gyrus had a negative relationship with the improvement in MoCA score (*r* = −0.669, *p* = 0.035).

**Table 4:** Significant correlations between the fcs and clinical assessment variables using a pearson correlation analysis.

Group	Time	FC variables	Clinical assessment	*r*(*p*)
TC-AOT	W12	Left TPJ and left MOG	∆ UPDRS-III	−0.698 (0.025)
	W12	Left pIPL and right insula	∆ BBS	0.644 (0.044)
	W12	∆ Left ATL and right SMG	∆ MMSE	0.670 (0.034)
TPT	W12	dmPFC and right FO	∆ Mini-BESTest	0.666 (0.035)
	W12	Left STS and right lingual	∆ MoCA	−0.669 (0.035)

∆ means change before and after rehabilitation (W12 – W0). TPJ, temporal parietal junction; MOG, middle occipital gyrus; pIPL, posterior inferior parietal lobe; ATL, anterior temporal lobe; SMG, supramarginal gyrus; dmPFC, dorsal medial prefrontal cortex; FO, frontal operculum; UPDRS-III, Unified Parkinson’s Disease Scale; MMSE, Mini-Mental Status Examination score; MoCA, Montreal Cognitive Assessment; BBS, Berg Balance Scale; Mini-BESTest, Mini Balance Evaluation Systems Test.

## 4. Discussion

This study investigated the effect of the TC-AOT intervention on brain FC changes in early-stage PD patients compared to a physical rehabilitation method. The resting-state DMN was used, and the correlations between changes in FCs and clinical scores were analyzed. The FCs between the brain regions that are involved in executive attention and motor learning, including the TPJ, SMG, IFG, and insula, were significantly increased after 12 weeks of TC-AOT training. The increased FCs were correlated to the improvements in UPDRS-III and BBS scores. Our results suggested that the TC-AOT intervention, which incorporates cognitive neural pathways in motor learning, has great potential benefits for early-stage PD.

A novel AOT intervention was proposed in the study, where continuous Tai Chi movements were first incorporated with AOT training. Agosta et al. have proved that the AOT combined with mobility exercise can increase brain activity in frontoparietal areas during fMRI tasks (Agosta et al., 2017). The TC-AOT protocol requires participants to observe, imagine, and imitate sequential movements, which involves more interplay between cognition and motor control. Recent studies have shown that modulation of the TPJ and SMG can influence motor attention performance for complex skilled actions (Saxe et al., 2004; Davis et al., 2018; Farina et al., 2020). Moreover, the IPL and IFG regions are critical for motor learning (Johnson-Frey et al., 2003; Buccino et al., 2004; Grafton et al., 2007). The insula plays a crucial role in the integration of sensory information for motor planning (Christopher et al., 2014; Huang et al., 2020). Consistent with these studies, we also observed increased FCs in the abovementioned brain areas after 12 weeks of rehabilitation. As shown in Table 4, the increased FC between left TPJ and left MOG was significantly related to the decrease in UPDRS-III scores, while the increased FC between left ATL and right SMG was associated with the improvement of cognitive function. The results demonstrated that the cognitive-motor interaction in the TC-AOT could promote brain plasticity and enhance motor control ability in PD patients (Herold et al., 2018).

The TC-AOT and TPT have different underlying mechanisms for PD rehabilitation. Significant time × group interactions were observed in FC changes in specific brain areas. The TC-AOT group exhibited increased FCs between the dmPFC and cerebellum crus1, right pIPL, and supramarginal cortex, as well as the TPJ to the region of the occipital and temporal gyrus, while the opposite trends were found in the TPT group (Figure 3). The temporoparietal junction, as well as the right pIPL and supramarginal cortex, are essential for visuospatial recognition, which may be related to the involvement of motion observation in the TC-AOT (Aracil-Bolaños et al., 2019). On the other hand, the PFC plays an important role in the regulation of behavior and cognition within the network (Friedman and Robbins, 2022). The strengthened cerebellar-prefrontal pathway could facilitate the transfer of sensorimotor information into ongoing cortical processing during goal-directed behaviors (Watson et al., 2014). It can be concluded that the TC-AOT has a unique advantage in improving cognitive functioning in PD associated with efficient motor learning.

The TPT performs less contribution to modulation in FCs of brain regions for early-stage PD patients. Only an increased FC between the dmPFC and the area of the right frontal operculum was observed (Figure 2F). Li et al. (2022) demonstrated that physical exercise could modify the plasticity of the frontal cortex. The increased FC was significantly correlated with improved balance ability. The intervention has also been regarded as a complementary option for PD management (Frazzitta et al., 2011; Bloem et al., 2015). However, in contrast with previous studies that revealed that physical exercise has a positive effect on cognitive function by influencing activation and metabolism of the frontal lobe circuit, there were no significant changes in the motor-cognitive pathway in the TPT group. Notably, we observed a decreased FC between the left STS and lingual gyrus, which was correlated with a declining trend in cognitive function, especially in visual perceptual integration (Pagonabarraga et al., 2013). Although both the TC-AOT and TPT groups exhibited improvements in motor and balance performance as well as quality of life, the TPT may not have a preventative effect on PD progression.

There are some limitations to be mentioned. First, the sample size was relatively small because it was a pilot study of a novel PD rehabilitation intervention. However, the calculation result of the *post-hoc* power analysis was 0.79, demonstrating that the statistical analysis and related conclusions were still effective in our study. Second, there were no significant differences in clinical assessments between the TC-AOT and TPT groups, which might be because the pen-and-paper tests may not be sensitive enough to detect subtle changes in PD symptoms. Quantitative measurement, especially for assessing motor automaticity ability, such as dual-task tests, should be considered in future studies.

Overall, the study was the first attempt to investigate the underlying mechanisms of a TC-AOT intervention for early-stage PD rehabilitation by analyzing resting-state fMRI based on the DMN compared to physical therapy. Both groups showed significant improvement in motor functions, enhancement in balance ability, and quality of life. However, increased FCs among the DMN nodes and several brain regions, including the frontal, insula, supramarginal, and occipital lobes, were observed and found to be significantly associated with cognitive and motor function improvement in the TC-AOT group. The proposed TC-AOT intervention demonstrated great potential in preventing cognitive decline and motor dysfunctions by promoting the interplay between cognition and motor control in early-stage PD patients.

## Data availability statement

The original contributions presented in the study are included in the article/Supplementary material, further inquiries can be directed to the corresponding authors.

## Ethics statement

The studies involving humans were approved by The Ethics Committee of Tianjin General Hospital. The studies were conducted in accordance with the local legislation and institutional requirements. The participants provided their written informed consent to participate in this study.

## Author contributions

LM, XZ, and DM: conception and design of the study. DW and YS: rehabilitation training and data collection. ZL and JZ: patient recruitment and clinical assessment. LM and DW: data interpretation and manuscript drafting. HL: critical manuscript revision for important intellectual content. XZ: clinical administration. DM: project administration. All authors approved the final version to be submitted.

## Footnotes

^1^ https://www.fil.ion.ucl.ac.uk/spm/

## References

[ref1] Aarsland D. Creese B. Politis M. Chaudhuri K. R. ffytche D. H. Weintraub D.. (2017). Cognitive decline in Parkinson disease. Nat. Rev. Neurol. 13, 217–231. doi: 10.1038/nrneurol.2017.27, PMID: 28257128 PMC5643027

[ref2] Agosta F. Gatti R. Sarasso E. Volonté M. Canu E. Meani A.. (2017). Brain plasticity in Parkinson’s disease with freezing of gait induced by action observation training. J. Neurol. 264, 88–101. doi: 10.1007/s00415-016-8309-7, PMID: 27778161

[ref3] Andrews-Hanna J. Reidler J. Huang C. Buckner R. (2010). Evidence for the default network's role in spontaneous cognition. J. Neurophysiol. 104, 322–335. doi: 10.1152/jn.00830.2009, PMID: 20463201 PMC2904225

[ref4] Aracil-Bolaños I. Sampedro F. Marín-Lahoz J. Horta-Barba A. Martínez-Horta S. Botí M.. (2019). A divergent breakdown of neurocognitive networks in Parkinson's disease mild cognitive impairment. Hum. Brain Mapp. 40, 3233–3242. doi: 10.1002/hbm.24593, PMID: 30938027 PMC6865605

[ref5] Ashburner J. (2007). A fast diffeomorphic image registration algorithm. NeuroImage 38, 95–113. doi: 10.1016/j.neuroimage.2007.07.007 17761438

[ref6] Ashburner J. Friston K. J. (2005). Unified segmentation. NeuroImage 26, 839–851. doi: 10.1016/j.neuroimage.2005.02.018 15955494

[ref7] Baggio H.-C. Segura B. Sala-Llonch R. Marti M.-J. Valldeoriola F. Compta Y.. (2015). Cognitive impairment and resting-state network connectivity in Parkinson's disease. Hum. Brain Mapp. 36, 199–212. doi: 10.1002/hbm.22622, PMID: 25164875 PMC6869118

[ref8] Bender R. Lange S. (2001). Adjusting for multiple testing—when and how? J. Clin. Epidemiol. 54, 343–349. doi: 10.1016/S0895-4356(00)00314-0, PMID: 11297884

[ref9] Bloem B. R. de Vries N. M. Ebersbach G. (2015). Nonpharmacological treatments for patients with Parkinson's disease. Mov. Disord. 30, 1504–1520. doi: 10.1002/mds.26363 26274930

[ref10] Bloem B. Marinus J. Dibble L. Nieuwboer A. Post B. Růžička E.. (2016). Measurement instruments to assess posture, gait, and balance in Parkinson's disease: critique and recommendations. Mov. Disord. 31, 1342–1355. doi: 10.1002/mds.26572, PMID: 26945525

[ref11] Bloem B. R. Okun M. S. Klein C. (2021). Parkinson's disease. Lancet 397, 2284–2303. doi: 10.1016/S0140-6736(21)00218-X 33848468

[ref12] Buccino G. Vogt S. Ritzl A. Fink G. R. Zilles K. Freund H.-J.. (2004). Neural circuits underlying imitation learning of hand actions: an event-related fMRI study. Neuron 42, 323–334. doi: 10.1016/S0896-6273(04)00181-3, PMID: 15091346

[ref13] Buckner R. L. Andrews-Hanna J. R. Schacter D. L. (2008). The brain's default network. Ann. N. Y. Acad. Sci. 1124, 1–38. doi: 10.1196/annals.1440.011 18400922

[ref14] Caligiore D. Mustile M. Fineschi A. Romano L. Piras F. Assogna F.. (2019). Action observation with dual task for improving cognitive abilities in Parkinson’s disease: A pilot study. Front. Syst. Neurosci. 13:7. doi: 10.3389/fnsys.2019.00007, PMID: 30804762 PMC6378302

[ref15] Caligiore D. Mustile M. Spalletta G. Baldassarre G. (2017). Action observation and motor imagery for rehabilitation in Parkinson's disease: a systematic review and an integrative hypothesis. Neurosci. Biobehav. Rev. 72, 210–222. doi: 10.1016/j.neubiorev.2016.11.005, PMID: 27865800

[ref16] Calleo J. Burrows C. Levin H. Marsh L. Lai E. York M. K. (2012). Cognitive rehabilitation for executive dysfunction in Parkinson's disease: application and current directions. Parkinson’s Dis. 2012:512892, 1–6. doi: 10.1155/2012/512892, PMID: 22135762 PMC3216311

[ref17] Cao W. Cao X. Hou C. Li T. Cheng Y. Jiang L.. (2016). Effects of cognitive training on resting-state functional connectivity of default mode, salience, and central executive networks. Front. Aging Neurosci. 8:70. doi: 10.3389/fnagi.2016.00070, PMID: 27148042 PMC4828428

[ref18] Chen K.-L. Xu Y. Chu A.-Q. Ding D. Liang X.-N. Nasreddine Z. S.. (2016). Validation of the Chinese version of montreal cognitive assessment basic for screening mild cognitive impairment. J. Am. Geriatr. Soc. 64, e285–e290. doi: 10.1111/jgs.14530, PMID: 27996103

[ref19] Christopher L. Koshimori Y. Lang A. E. Criaud M. Strafella A. P. (2014). Uncovering the role of the insula in non-motor symptoms of Parkinson’s disease. Brain 137, 2143–2154. doi: 10.1093/brain/awu084, PMID: 24736308 PMC4107733

[ref20] Chumbley J. Worsley K. Flandin G. Friston K. (2010). Topological FDR for neuroimaging. NeuroImage 49, 3057–3064. doi: 10.1016/j.neuroimage.2009.10.090, PMID: 19944173 PMC3221040

[ref21] Davis S.W. Wing E.A. Cabeza R. (2018). Chapter 27 – contributions of the ventral parietal cortex to declarative memory. Amsterdam Elsevier. 10.1016/B978-0-444-63622-5.00027-9 29519478

[ref22] DeLong M. R. Wichmann T. (2007). Circuits and circuit disorders of the basal ganglia. Arch. Neurol. 64, 20–24. doi: 10.1001/archneur.64.1.20 17210805

[ref23] Farina E. Borgnis F. Pozzo T. (2020). Mirror neurons and their relationship with neurodegenerative disorders. J. Neurosci. Res. 98, 1070–1094. doi: 10.1002/jnr.24579, PMID: 31975553

[ref24] Ferrazzoli D. Ortelli P. Cucca A. Bakdounes L. Canesi M. Volpe D. (2020). Motor-cognitive approach and aerobic training: a synergism for rehabilitative intervention in Parkinson's disease. Neurodegenerat. Dis. Manag. 10, 41–55. doi: 10.2217/nmt-2019-0025, PMID: 32039653

[ref25] Ferrazzoli D. Ortelli P. Madeo G. Giladi N. Petzinger G. M. Frazzitta G. (2018). Basal ganglia and beyond: the interplay between motor and cognitive aspects in Parkinson’s disease rehabilitation. Neurosci. Biobehav. Rev. 90, 294–308. doi: 10.1016/j.neubiorev.2018.05.007, PMID: 29733882

[ref26] Florio T. (2018). The basal ganglia: more than just a switching device. CNS Neurosci. Ther. 24, 677–684. doi: 10.1111/cns.12987, PMID: 29879292 PMC6490066

[ref27] Folstein M. Folstein S. E. McHugh P. (1975). “Mini-mental state”. A practical method for grading the cognitive state of patients for the clinician. J. Psychiatr. Res. 12, 189–198. doi: 10.1016/0022-3956(75)90026-6, PMID: 1202204

[ref28] Franchignoni F. Velozo C. (2005). Use of the berg balance scale in rehabilitation evaluation of patients with Parkinson’s disease. Arch. Phys. Med. Rehabil. 86, 2225–2226. doi: 10.1016/j.apmr.2005.09.006 16271578

[ref29] Frazzitta G. Bertotti G. Riboldazzi G. Turla M. Uccellini D. Boveri N.. (2011). Effectiveness of intensive inpatient rehabilitation treatment on disease progression in parkinsonian patients: a randomized controlled trial with 1-year follow-up. Neurorehabil. Neural Repair 26, 144–150. doi: 10.1177/1545968311416990, PMID: 21844282

[ref30] Friedman N. P. Robbins T. W. (2022). The role of prefrontal cortex in cognitive control and executive function. Neuropsychopharmacology 47, 72–89. doi: 10.1038/s41386-021-01132-0, PMID: 34408280 PMC8617292

[ref31] Goetz C. G. (2003). The unified Parkinson's disease rating scale (UPDRS): status and recommendations. Mov. Disord. 18, 738–750. doi: 10.1002/mds.10473 12815652

[ref32] Grafton S. T. de C. Hamilton A. F. (2007). Evidence for a distributed hierarchy of action representation in the brain. Hum. Mov. Sci. 26, 590–616. doi: 10.1016/j.humov.2007.05.009, PMID: 17706312 PMC2042582

[ref33] Herold F. Hamacher D. Schega L. Müller N. G. (2018). Thinking while moving or moving while thinking – concepts of motor-cognitive training for cognitive performance enhancement. Front. Aging Neurosci. 10:228. doi: 10.3389/fnagi.2018.00228, PMID: 30127732 PMC6089337

[ref34] Hoehn M. Yahr M. (2001). Hoehn MM, Yahr MD. Parkinsonism: onset, progression and mortality. Neurology 17, 427–426. doi: 10.1212/WNL.17.5.427 6067254

[ref35] Hou Y. Yang J. Luo C. Song W. Ou R. Liu W.. (2016). Dysfunction of the default mode network in drug-Naïve Parkinson’s disease with mild cognitive impairments: a resting-state fMRI study. Front. Aging Neurosci. 8:247. doi: 10.3389/fnagi.2016.00247, PMID: 27833548 PMC5080293

[ref36] Huang P. Guan X. Guo T. Zeng Q. Xuan M. Gu Q.. (2020). Damaged insula network contributes to depression in Parkinson’s disease. Front. Psych. 11, –119. doi: 10.3389/fpsyt.2020.00119, PMID: 32210851 PMC7076072

[ref37] Johansson M. E. Cameron I. G. M. Van der Kolk N. M. de Vries N. M. Klimars E. Toni I.. (2022). Aerobic exercise alters brain function and structure in Parkinson's disease: a randomized controlled trial. Ann. Neurol. 91, 203–216. doi: 10.1002/ana.26291, PMID: 34951063 PMC9306840

[ref38] Johnson-Frey S. H. Maloof F. R. Newman-Norlund R. Farrer C. Inati S. Grafton S. T. (2003). Actions or hand-object interactions? Human inferior frontal cortex and action observation. Neuron 39, 1053–1058. doi: 10.1016/S0896-6273(03)00524-5 12971903

[ref39] King L. Mancini M. Smulders K. Harker G. Lapidus J. Ramsey K.. (2020). Cognitively challenging agility boot camp program for freezing of gait in Parkinson disease. Neurorehabil. Neural Repair 34, 417–427. doi: 10.1177/1545968320909331, PMID: 32249668 PMC7217755

[ref40] Lan C. Chen S.-Y. Lai J.-S. Wong A. M.-K. (2013). Tai chi Chuan in medicine and health promotion. Evid. Based Complement. Alternat. Med. 2013:502131, 1–17. doi: 10.1155/2013/502131, PMID: 24159346 PMC3789446

[ref41] Li J. Guo J. Sun W. Mei J. Wang Y. Zhang L.. (2022). Effects of exercise on Parkinson’s disease: a meta-analysis of brain imaging studies. Front. Hum. Neurosci. 16:796712. doi: 10.3389/fnhum.2022.796712, PMID: 35250515 PMC8889068

[ref42] Li F. Harmer P. Fitzgerald K. Eckstrom E. Stock R. Galver J.. (2012). Tai Chi and postural stability in patients with Parkinson's disease. N. Engl. J. Med. 366, 511–519. doi: 10.1056/NEJMoa1107911, PMID: 22316445 PMC3285459

[ref43] Maidan I. Rosenberg-Katz K. Jacob Y. Giladi N. Hausdorff J. M. Mirelman A. (2017). Disparate effects of training on brain activation in Parkinson disease. Neurology 89, 1804–1810. doi: 10.1212/WNL.0000000000004576, PMID: 28954877

[ref44] Nasreddine Z. Phillips N. Bédirian V. Charbonneau S. Whitehead V. Collin I.. (2005). The Montreal Cognitive Assessment, MoCA: a brief screening tool for mild cognitive impairment. J. Am. Geriatr. Soc. 53, 695–699. doi: 10.1111/j.1532-5415.2005.53221.x, PMID: 15817019

[ref45] Opdebeeck C. Martyr A. Clare L. (2016). Cognitive reserve and cognitive function in healthy older people: a meta-analysis. Aging Neuropsychol. Cognit. 23, 40–60. doi: 10.1080/13825585.2015.1041450, PMID: 25929288

[ref46] Pagonabarraga J. Corcuera-Solano I. Vives-Gilabert Y. Llebaria G. García-Sánchez C. Md B.. (2013). Pattern of regional cortical thinning associated with cognitive deterioration in Parkinson’s disease. PLoS One 8, –e54980. doi: 10.1371/journal.pone.0054980, PMID: 23359616 PMC3554657

[ref47] Porto F. H. G. Coutinho A. M. de Souza Duran F. L. de Sá Pinto A. L. Gualano B. Buchpiguel C. A.. (2018). Aerobic training modulates salience network and default mode network metabolism in subjects with mild cognitive impairment. NeuroImage 19, 616–624. doi: 10.1016/j.nicl.2018.05.002, PMID: 29984169 PMC6031093

[ref48] Sarasso E. Agosta F. Piramide N. Gardoni A. Canu E. Leocadi M.. (2021). Action observation and motor imagery improve dual task in Parkinson's disease: a clinical/fMRI study. Mov. Disord. 36, 2569–2582. doi: 10.1002/mds.28717, PMID: 34286884

[ref49] Saxe R. Carey S. Kanwisher N. (2004). Understanding other minds: linking developmental psychology and functional neuroimaging. Annu. Rev. Psychol. 55, 87–124. doi: 10.1146/annurev.psych.55.090902.142044, PMID: 14744211

[ref50] Snyder A. Z. Raichle M. E. (2012). A brief history of the resting state: the Washington University perspective. NeuroImage 62, 902–910. doi: 10.1016/j.neuroimage.2012.01.044, PMID: 22266172 PMC3342417

[ref51] Soares J. Magalhaes R. Moreira P. Sousa A. Ganz E. Sampaio A.. (2016). A Hitchhiker's guide to functional magnetic resonance imaging. Front. Neurosci. 10:515. doi: 10.3389/fnins.2016.00515, PMID: 27891073 PMC5102908

[ref52] Thibes R. Novaes N. Lucato L. Campanholo K. Melo L. Leite C.. (2017). Altered functional connectivity between precuneus and motor systems in Parkinson's disease patients. Brain Connect. 7, 643–647. doi: 10.1089/brain.2017.0534, PMID: 29065697

[ref53] Watson T. Becker N. Apps R. Jones M. (2014). Back to front: cerebellar connections and interactions with the prefrontal cortex. Front. Syst. Neurosci. 8:4. doi: 10.3389/fnsys.2014.00004, PMID: 24550789 PMC3912388

[ref54] Whitfield-Gabrieli S. Nieto-Castanon A. (2012). Conn: a functional connectivity toolbox for correlated and anticorrelated brain networks. Brain Connect. 2, 125–141. doi: 10.1089/brain.2012.0073, PMID: 22642651

[ref55] Worsley K. J. Marrett S. Neelin P. Vandal A. C. Friston K. J. Evans A. C. (1996). A unified statistical approach for determining significant signals in images of cerebral activation. Hum. Brain Mapp. 4, 58–73. doi: 10.1002/(SICI)1097-0193(1996)4:1<58::AID-HBM4>3.0.CO;2-O, PMID: 20408186

