# Supplementary material for: Enhanced brain functional connectivity and activation after 12-week Tai Chi-based action observation training in patients with Parkinson’s disease
Source: Front Aging Neurosci. 2023 Oct 10;15:1252610. doi: 10.3389/fnagi.2023.1252610 (PMC10595151; doi:10.3389/fnagi.2023.1252610)
Supplement: Supplementary file 1 [file Table_1.DOCX]

Supplementary Material

Enhanced Brain Functional Connectivity and Activation after 12-weeks Tai Chi Based Action Observation Training in Patients with Parkinson's Disease

**Lin Meng, Deyu Wang, Yu Shi, Zhuo Li, Jinghui Zhang, Hanna Lu, Xiaodong Zhu, and Dong Ming**

**Correspondence:** Corresponding Author:

Lin Meng [linmeng@tju.edu.cn](mailto:linmeng@tju.edu.cn)

Dong Ming richardming@tju.edu.cn

# Supplementary Figures and Tables

**Table S1**

*Default modes network nodes for seed based functional connectivity analysis­­*

| Seed region | MNI coordinate (x, y, z) | | |
| --- | --- | --- | --- |
| Anterior medial prefrontal cortex | -8 | 56 | 14 |
| Dorsal medial prefrontal cortex | -8 | 50 | 34 |
| Ventral medial prefrontal cortex | -2 | 44 | -12 |
| Superior frontal gyrus | -8 | 20 | 62 |
| Left inferior frontal gyrus | -42 | 26 | -14 |
| Right inferior frontal gyrus | 50 | 32 | -6 |
| Left posterior inferior parietal lobule | -50 | -60 | 28 |
| Right posterior inferior parietal lobule | 58 | -60 | 28 |
| Precuneus | -2 | -60 | 50 |
| Posterior cingulate cortex | -2 | -48 | 28 |
| Left anterior temporal lobe | -52 | -10 | -20 |
| Right anterior temporal lobe | 52 | -4 | -16 |
| Left superior temporal sulcus | -60 | -28 | -4 |
| Right superior temporal sulcus | 50 | -36 | 4 |
| Left temporal parietal junction | -44 | -52 | 22 |
| Right temporal parietal junction | 44 | -58 | 18 |
| Left hippocampal formation | -26 | -8 | -24 |
| Right hippocampal formation | 24 | -14 | -22 |

*Note:* MNI = Montreal Neurological Institute.
